# Effectiveness of chemotherapy using bortezomib combined with homoharringtonine and cytarabine in refractory or relapsed acute myeloid leukemia: a phase II, multicenter, prospective clinical trial

**DOI:** 10.3389/fonc.2023.1142449

**Published:** 2023-08-18

**Authors:** Chengtao Zhang, Da Gao, Xiaohong Wang, Xiuli Sun, Yan Yan, Yan Yang, Jingjing Zhang, Jinsong Yan

**Affiliations:** ^1^ Department of Hematology, Liaoning Key Laboratory of Hematopoietic Stem Cell Transplantation and Translational Medicine, Liaoning Medical Center for Hematopoietic Stem Cell Transplantation, The Second Hospital of Dalian Medical University, Dalian, China; ^2^ Department of Hematology, The Affiliated Hospital of Inner Mongolia Medical University, Hohhot, Inner Mongolia, China; ^3^ Department of Hematology, The ChaoYang Central Hospital, Liaoning, China; ^4^ Department of Hematology, The First Affiliated Hospital of Dalian Medical University, Dalian, China; ^5^ Department of Hematology, Bayannur Hospital, Bayannur, Inner Mongolia, China; ^6^ Blood Stem Cell Transplantation Institute of Dalian Medical University, Dalian, China; ^7^ Pediatric Oncology and Hematology Center of the Second Hospital of Dalian Medical University, Dalian, China

**Keywords:** acute myeloid leukemia, refractory and relapsed, bortezomib, homoharringtonine, FLT3 mutation, induction

## Abstract

**Background:**

Refractory/relapsed acute myeloid leukemia (R/R AML) has unsatisfactory outcomes even after allogeneic hematopoietic stem cell transplantation. Long-term survival is mainly influenced by complete remission (CR) rates after induction therapies.

**Objectives:**

To investigate CR/CR with incomplete hematologic recovery (CRi) rates and adverse events with a new induction therapy (bortezomib, homoharringtonine, and cytarabine [BHA]) for patients with R/R AML.

**Methods:**

We enrolled 21 patients with R/R AML (median age, 42 [range, 30–62] years), who received BHA for remission induction (bortezomib, 1.3 mg/m^2^/day on days 1 and 4; homoharringtonine, 4 mg/m^2^/day for 5 days, and cytarabine, 1.5 g/m^2^/day for 5 days). CR and adverse events were assessed.

**Results:**

After one course of BHA, the CR/CRi and partial remission rates were 38.1% and 14.3%, respectively, with an overall response rate (ORR) of 52.4% in 21 patients. 9 of 21 patients harbored FLT3-ITD or FLT3-TKD mutations, and achieved either CR/CRi or ORR of 66.7% (*P*=0.03) by comparison with that in R/R AML without FLT3 mutation. After induction therapy, consolidation chemotherapy or allogeneic hematopoietic stem cell transplantation led to a one-year overall survival of 27.8% in all patients. One-year relapse-free survival was 50% in 8 patients who had achieved CR/CRi after one course of BHA. During induction, non-hematologic adverse events (grade 3/4) commonly were infection (90.5%), hypokalemia (14.4%), hypocalcemia (14.3%), and mucositis (9.5%). In patients achieving CR, the median time to neutrophil count >0.5×10^9^/L and time to platelet count >20×10^9^/L were 15 (13–17) days and 13 (13–18) days, respectively.

**Conclusion:**

BHA chemotherapy regimen was safe and tolerable to serve as an induction therapy for R/R AML, particularly with FLT3 mutation. The higher CR/CRi rate will give a clue to determine a potentialeffectiveness of BHA for AML patients carrying FLT3 mutation in a further investigation.

**Clinical trial registration:**

https://www.chictr.org.cn/, identifier ChiCTR2000029841.

## Introduction

The outcome of patients with relapsed or refractory acute myeloid leukemia (R/R AML) is not satisfactory, with a 5-year overall survival (OS) rate of approximately 10% (1).Primary refractory AML occurs in 10–40% of newly diagnosed patients with AML (2). Relapsed AML occurs in 40–50% of patients who achieved complete remissions (CR) (3, 4). For patients with R/R AML, a successful CR achieved with induction therapy followed by allogeneic hematopoietic stem cell transplantation (HSCT) is considered a preferable option because its 3-year OS rate can reach up to 40% (5, 6), whereas without achieving CR in the induction therapy, the 3-year OS is even with the addition of HSCT only 19% (7).Therefore, CR plays a critical role in the long-term OS of patients with R/R AML. However, the response to induction chemotherapy remains varied with CR rates of 18–61.7% when utilizing remission induction regimens, such as high-dose cytarabine (HiAra-C) (8); fludarabine, cytarabine, and granulocyte colony-stimulating factor (FLGA), or idarubicin combine into FLAG(FLAG-IDA) (9) (10); cladribine, cytarabine and granulocyte colony-stimulating factor (CLAG) (9, 11); mitoxantrone, etoposide and cytarabine (MEC) (12), and demethylating agents combined with venetoclax (13). CLAG regimen can reach a CR rate of 61.7%, which is numerically higher than that with FLAG of 48.7%, however, there were no difference of overall survival between FLAG and CLAG (9). In recent years mitoxantrone added into CLAG (CLAG-M) was used to treat R/R AML and high-grade myeloid neoplasm, yielding a CR rate of 60%, and 1-year OS of 44% at most (14), however due to its strong myelosuppression, it presented a higher risk to infectious complications and invasive fungal infection, which was a primary or contributing cause of death in 59% patients (15, 16). Therefore, new chemotherapy combinations should be designed and verified for R/R AML.

Homoharringtonine is usually incorporated into conventional regimens such as DA or IA (cytarabine with daunorubicin or idarubicin) as induction regimen for newly diagnosed AML in China (17, 18). Jin et al. (18) compared the results in young people with *de-novo* AML treated with homoharringtonine, cytarabine, and aclarubicin (HAA) or daunorubicin plus cytarabine (DA). The CR rates were 73% and 61% (*P*=0.0108), and the 3-year event-free survival rates were 35.4% and 23.1% (*P*=0.0023), respectively. As for R/R AML, or carrying FLT3 mutation, homoharringtonine usually is combined with other therapeutic agents, for instance, homoharringtonine added into venetoclax and cytarabine yielded a CR of 70.8% (19), into DAC-HAA for pediatric R/R AML yielded CR of 77.4% (20), and into FLT3-mutation inhibitor yielded a inhibitory effect in leukemia cell lines (21, 22). Homoharringtonine at a lower dosage may play an adjuvant role for promoting apoptosis of leukemia cells, which may improve the effects against R/R AML leukemia. However, the precise contribution of homoharringtonine, its accurate dosage, and treatment duration remained unclear.

It has been reported elsewhere that homoharringtonine is effective and safe for R/R AML at a dosage of 4-5mg/m^2^ for consecutive 7 days, when combined with cytarabine(HA) (23). Even HA regimen only achieved a CR rate of 22.7% for treating elderly R/R AML (23), however, if other chemotherapeutic agent is added into HA, then new combination regimen may improve its treatment effect for R/R AML. It is well known that bortezomib, a proteasome inhibitor, approved by the FDA for multiple myeloma (24) and mantle cell lymphoma (25), has shown antitumor effects in AML cell lines U937 and KG1, as well as in leukemic blasts from patients with AML (26, 27). It has been reported that bortezomib added to DA regimen can achieve a CR rate of 65% in patients aged 60–75 years with newly diagnosed AML (28). However, the effectiveness of bortezomib combined with HA for treating R/R AML remains unknown.

Here, we designed a BHA regimen (bortezomib added into HA regimen) for patients with R/R AML and to investigate its CR rate and safety in induction therapy. Furthermore, the OS of patients with R/R AML was analyzed to assess the effectiveness of BHA regimen.

## Methods

### Study design and participants

This phase II, multicenter, single-arm study was registered at the Chinese Clinical Trial Registry (ChiCTR2000029841) and was conducted in five institutions in China). All patients aged 18–65 years and diagnosed with refractory or relapsed AML were considered eligible for this study. The enrolled patients were required to meet the following criteria: Eastern Cooperative Oncology Group performance status (ECOG) score ≤3. The exclusion criteria were as follows: patients with acute promyelocytic leukemia, with extramedullary or central nervous infiltration, hepatic or renal insufficiency, uncontrolled infection, receiving other treatments for leukemia, allergy to any of the study drugs, and refusal to participate in this study. All patients have provided informed consent before enrollment and chemotherapy. All performance and the treatment protocol were approved by institutional review boards and the ethics committee in the Second Hospital of Dalian Medical University. The study was in accordance with the ethical standards of Declaration of Helsinki (1975) and its later amendments.

### Treatment protocol

All patients underwent a full physical examination, blood routine, liver function, kidney function, blood glucose, electrocardiogram, and chest CT before each course of induction and consolidation chemotherapy. Bone marrow aspirate and immunophenotyping by flow cytometry were performed to assess treatment response at the 21st through 28th days after each chemotherapy course.

Patients with R/R AML received BHA as an induction therapy. Patients who had achieved CR or CR with incomplete hematologic recovery (CRi) received further consolidation therapy or allogeneic HSCT if suitable donor was available.

BHA consisted of bortezomib, homoharringtonine and cytarabine. Bortezomib was intravenously administered at a dose of 1.3 mg/m^2^ of body surface area on days 1 and 4, 2 hours before homoharringtonine. Homoharringtonine (4 mg/m^2^) was continuously infused over 4 hours once daily for 5 consecutive days. Cytarabine (1.5 g/m^2^) was continuously infused over 3 hours once daily for 5 consecutive days.

After induction therapy, patients who had achieved CR or CRi further received allogeneic HSCT or three to four courses of consolidation chemotherapy. Consolidation chemotherapy regimens included cytarabine combined with idarubicin or HA, with detailed dosages as follows: cytarabine (2 g/m^2^) twice daily on days 1, 3, and 5 in each consolidation course, idarubicin (12 mg/m^2^) continuously infused over 2 hours once daily for 3 days, or homoharringtonine (4 mg/m^2^) once daily continuously infused over 4 hours once daily for 5 days. For patients who had achieved partial remission (PR), one more course of BHA regimen was used, but for those who did not tolerate a second course of BHA, other salvage chemotherapy regimens were chosen at the physician’s discretion. If patients achieved CR or CRi after the second course of BHA or the salvage chemotherapy, then these patients continued consolidation chemotherapy with cytarabine combined with idarubicin or HA mentioned above.

When patients achieved non-remission (NR) with one course of BHA, or PR with the second course of BHA or other regimens at the physician’s discretion, they were further treated with salvaged allogeneic HSCT (7) or other chemotherapies such as CLAG (11), decitabine added into cytarabine, aclacinomycin and granulocyte colony-stimulating factor (CAG) (29), in accordance with the NCCN Clinical Practice Guidelines in Oncology: Acute Myeloid Leukemia (Version 3.2019) (30). Infection prophylaxis including antibacterial, anti-pneumocystis, antifungal, and antiviral agents were given according to the relevant guidelines (31–33).

### Endpoints and definitions

CR/CRi was the primary endpoint of this study after induction therapy with one course of BHA. The secondary endpoints were safety evaluation and 1-year estimated OS.

According to the 2017 European LeukemiaNet (ELN) (6) Response criteria were as follows: (1) CR: bone marrow blasts <5%, absence of circulating blasts and blasts with Auer rods, absence of extramedullary disease, absolute neutrophile count 1.0×10^9^/L, platelet count ≥100×10^9^/L; (2) CRi: all CR criteria except for residual neutropenia (<1.0×10^9^/L) or thrombocytopenia (<100×10^9^/L); (3) PR: all hematologic criteria of CR, decrease in bone marrow blast percentage to 5-25%, and decrease in pretreatment bone marrow blast percentage by at least 50%; (4) NR: patients not categorized as CR, CRi, or PR; (5) overall response rate (ORR): the proportion of patients with the best overall response including CR, CRi, and PR; (6) relapse: bone marrow blasts ≥5%, reappearance of blasts in the blood, or development of extramedullary disease; and (7) primary refractory: failure to attain CR following at least two courses of intensive induction therapy.

In patients who experienced CR, the time to neutrophil recovery was measured from day 1 of induction treatment until the first day of 3 consecutive days that the absolute neutrophil count was ≥0.5×10^9^/L, and the time to platelet recovery was measured from day 1 of induction until the platelet count was ≥100×10^9^/L. Adverse events were assessed according to the National Cancer Institute Common Terminology Criteria for Adverse Events version 5.0 (34).

### Statistical analysis

This study was followed up until December 1, 2022. The distributions and frequencies of patients’ characteristics were reported using descriptive statistics. OS was defined for all patients, measured from the date of entry into the clinical trial to the date of death from any cause or to the date of the last follow-up. Relapse-free survival (RFS) was defined only for patients achieving CR or CRi, measured from the date of achievement of remission until the date of relapse or death from any cause; patients not known to have relapsed or died at last follow-up were censored on the date they were last examined (6). Between two groups, differences among continuous and categorical variables were compared using the Mann–Whitney U test and chi-square test, respectively. OS and RFS were estimated using Kaplan–Meier survival analysis. All analyses were performed using SPSS statistics version 27.0 (SPSS Inc., Chicago, IL, USA). *P*<0.05 was considered statistically significant.

## Results

### Patients

As shown in **Table 1**, 21 eligible patients with R/R AML including 13 men and 8 women were recruited for this study from May 2019 through March 2022. These patients with a median age of 42 (range, 30–62) years encompassed 6 primarily refractory and 15 relapsed AMLs. The 15 relapsed AMLs consisted of 13 patients with a first time of relapse and 2 with a second time of relapse. The cytogenetic assay showed 7 patients had normal karyotypes, 7 complex karyotypes, 2 t (8;21) (q22;q22.1), 1 a monosomal karyotype, and the remaining 4 had other karyotypes.

**Table 1:** Patients’ characteristics.

Characteristics	*n*=21
Sex, *n* (%)	
Male	13 (61.9)
Female	8 (38.1)
Age (years)	
Median	42
Range	30–62
FAB classification, *n* (%)	
M0	1 (4.8)
M2	8 (38.1)
M4	3 (14.3)
M5	9 (42.8)
Disease, *n* (%)	
*De novo*	19 (90.5)
Secondary to myelodysplastic syndrome	2 (9.5)
Disease status, *n* (%)	
Primary refractory	6 (28.6)
Relapse	15 (71.4)
First relapse	13 (61.9)
Second relapse	2 (9.5)
ECOG performance status, *n* (%)	
0	1 (4.8)
1	10 (47.6)
2	7 (33.3)
3	3 (14.3)
Karyotype, *n* (%)	
Normal	7 (33.3)
Abnormal	14 (66.7)
t (8;21) (q22;q22.1); RUNX1-RUNX1T1	2 (9.5)
Complex karyotype^a^	7 (33.3)
Monosomal karyotype^b^	1 (4.8)
Others	4 (19.1)
Molecular mutations, *n* (%)	
FLT3 mutation^c^	9 (42.8)
FLT3-ITD	6(28.5)
FLT3-TKD	2(9.5)
FLT3-ITD plus FLT3-TKD	1(4.8)
NPM1	7 (33.3)
DNMT3A	4 (19.1)
ASXL1	4 (19.1)
RUNX1	2 (9.5)
CEBPA	2 (9.5)
TP53	1 (4.8)
Percentage of blasts in the bone marrow, *n* (%)	
5–9%	3 (14.3)
10–19%	6 (28.5)
>20%	12 (57.1)
White blood cell count (×10^9^/L) Median (range)	2.9 (0.2–113.2)
Hemoglobin (g/dL) Median (range)	7.8 (2.4–11.9)
Platelet count (×10^9^/L) Median (range)	33 (3–291)
Median courses prior to chemotherapy Median (range)	2 (1–12)

ECOG, Eastern Cooperative Oncology Group.

a Three or more unrelated chromosome abnormalities in the absence of one of the WHO-designated recurring translocations or inversions.

b Presence of one single monosomy (excluding loss of X or Y) in association with at least one additional monosomy or structural chromosome abnormality (excluding core-binding factor AML).

c FLT3 mutation consisted of FLT3-ITD, FLT3-ITD concomitant with FLT3-TKD, FLT3-TKD.

Targeted next-generation sequencing showed that 9 patients harbored FLT3 mutations, consisting of 6 FLT3-ITD, 1 FLT3-ITD concomitant with FLT3-TKD, and 2 FLT3-TKD, moreover, some patients harbored NPM1, DNMT3A, ASXL1, RUNX1, CEBPA, or TP53 mutations. The karyotyping results and genetic mutations are listed in **Supplementary T** **able S1**.

When receiving induction therapy, the median white blood cell count was 2.9 (0.2–113.3) ×10^9^/L, the median hemoglobin concentration 7.8 (2.4–11.9) g/dL, the median platelet counts 22 (9–291) ×10^9^/L, and blast cell percentage in the bone marrow 23.0% (5.5–92.0%). The median time prior to induction therapy was 2 (1–12) months.

### Response

ORR was 52.4% (11/21), and CR/CRi rate was 38.1% (8/21) in patients with R/R AML treated using the BHA regimen. In 9 patients with FLT3 mutations, both ORR and CR/CRi rates were 66.7%.

In detail, 11/21 patients (52.4%) showed good responses, including 5 CR (23.8%), 3 CRi (14.3%), and 3 PR (14.3%). Among the three PR patients, 1 was repeatedly treated with BHA regimen, and then successfully achieved CR; unfortunately, he refused further treatments; 1 received decitabine combined with CAG chemotherapy; and the remaining1 received salvage allogeneic transplantation. Among 6 refractory and 15 relapsed AMLs, the ORRs were 50% (3/6) and 53.3% (8/15, *P*=0.77), respectively; and the CR/CRi rates were 33.3% (2/6) and 40% (6/15, *P*=0.30), respectively.

As showed in **Table 2**, 12 out of 21 patients have no FLT3 mutation and 9 carried FLT3 mutation. Strikingly, only 2 achieved CR (16.7%) and 3 PR (25%) in 12 patients without FLT3 mutation, but 6 achieved CR/CRi (66.7%) in 9 patients with FLT3 mutation. The CR/CRi rate improved significantly in patients with FLT3 mutation after treatment with BHA (p=0.03). Before entry of this trial, sorafenib-contained chemotherapy regimens were utilized in 5 FLT3-mutated AML patients, but they all finally developed into R/R AML. Interestingly, successful achievement of CR/CRi treated with BHA in 6 of 9 FLT3-mutated R/R AML indicated that BHA was effective as a salvage therapy even when sorafenib was of ineffective to R/R AML with FLT3 mutation. Notably, the remaining 3 patients had no response to BHA, who included 2 patients carried two types of concomitant FLT3-ITD insertion mutations and 1 with a single FLT3-ITD mutation combined with two DNMT3A mutations (**Supplementary T** **able S2**).

**Table 2:** Outcomes of one course BHA in 21 patients with refractory/relapsed AML.

	Patients (*n*)	CR (%)	CRi (%)	PR (%)	ORR (%)
	21	5 (23.8)	3 (14.3)	3 (14.3)	11 (52.4)
Primary refractory	6	1 (16.7)	1 (16.7)	1 (16.7)	3 (50.0)
Relapse	15	4 (26.7)	2 (13.3)	2 (13.3)	8 (53.3)
FLT3 mutations	9	6 (66.7)			6 (66.7)
FLT3-ITD	6	3			3
FLT3-TKD	2	2			2
FLT3-ITD plus FLT3-TKD	1	1			1
Wild-type FLT3	12	2 (16.7)		3 (25.0)	5 (41.7)

AML, acute myeloid leukemia; BHA, bortezomib, homoharringtonine, and cytarabine; CR, complete remission; CRi, CR with incomplete hematologic recovery; ORR, overall response rate; PR, partial remission.

After completion of the BHA 9 patients received haploidentical bone marrow transplants as a salvage option (35), and 12 patients Received the subsequent treatments: intermediate-dose cytarabine and idarubicin (36), venetoclax plus azacytidine (37), decitabine with CAG (29), or palliative support (38), in accordance with the NCCN Clinical Practice Guidelines in Oncology: Acute Myeloid Leukemia (**Supplementary Figure S1**) (30).

### Adverse events

**Table 3** summarizes the major adverse events. During induction in 21 patients, the most common adverse event was an infection. Infection-related symptoms were febrile neutropenia (90.5%), pneumonia (33.3%), sepsis (9.5%), and catheter-related infections (4.8%). Other common non-hematologic adverse events of any grade included hypoproteinemia (81.0%), nausea (57.1%), and hypokalemia (52.3%). Common non-hematologic adverse events of grade 3/4 were infection (90.5%), hypokalemia (14.3%), hypocalcemia (14.3%), and mucositis (9.5%). Three patients (14.3%) developed grade 1/2 peripheral neuritis, but no patient developed grade 3/4 damage to the nervous system. The chemotherapy was generally well tolerated, and no chemotherapy-related deaths occurred. The 30-day mortality was 4.8%.

**Table 3:** Treatment-related non-hematologic adverse events.

AE*	Any grade AE, *n* (%)	Grade 3/4 AE, *n* (%)
Infections	19 (90.5)	19 (90.5)
Hypoalbuminemia	17 (81.0)	0
Nausea	12 (57.1)	0
Hypokalemia	11 (52.3)	3 (14.3)
Hypocalcemia	10 (47.6)	3 (14.3)
Hepatic dysfunction	8 (38.1)	1 (4.8)
Vomiting	5 (23.8)	0
Abdominal pain	4 (19.1)	0
Diarrhea	4 (19.1)	0
Peripheral sensory neuropathy	3 (14.3)	0
Mucositis	2 (9.5)	2 (9.5)
Abdominal distension	1 (4.8)	0
Arrhythmia	1 (4.8)	1 (4.8)
Angina	1 (4.8)	0
Hyponatremia	1 (4.8)	1 (4.8)
Allergic dermatitis	1 (4.8)	0
DIC	1 (4.8)	0

AE, adverse event; DIC, disseminated intravascular coagulation.

*AEs were assessed based on the CTCAE (NCI Common Terminology Criteria for Adverse Events) version 5.0.

With induction therapy, severe neutropenia and thrombocytopenia occurred in all patients. The median white blood cell count decreased to 0.11 (0.02–0.83) ×10^9^/L, and the median platelet decreased to 11 (3–16) ×10^9^/L. In patients achieving CR, the median time to neutrophil count >0.5×10^9^/L and time to platelet count >20×10^9^/L was 15 (13–17) days and 13 (13–18) days, respectively, from the start of chemotherapy.

### Survival analysis

**Figure 1:**
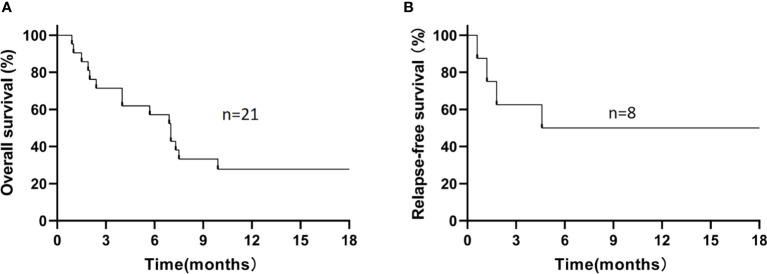
The 1-year estimated overall survival in all patients **(A)** and relapse-free survival in patients who had achieved CR/CRi **(B)**. **(A)** The 1-year estimated OS rate was 27.8% in all patients. **(B)** Among 8 patients who had achieved CR/CRi, the 1-year estimated RFS rate was 50%.

For the 21 enrolled patients, the median follow-up time was 7.0 (0.9–30.1) months, the 1-year estimated OS rate was 27.8% (**Figure 1A**), and the median OS was 7.0 (0.9–30.1) months. The 1-year estimated OS rate was 44.4% in FLT3-mutated AML but only 16.7% without FLT3 mutation (*P*=0.12).

During the follow-up, 6/21 patients (28.6%) survived, and 15/21 patients (71.4%) died. The causes of death in these 15 patients included disease progression (*n*=5), severe infection (*n*=4, including severe pneumonia [*n*=3] and sepsis [*n*=1]), relapse (*n*=4), and cerebral hemorrhage (*n*=2; **Supplementary Figure S1**). Among those 8 patients who had achieved CR/CRi, the 1-year estimated OS rate was 62.5%, with a median OS time of 9.3 (1.9–30.1) months, and the 1-year estimated RFS rate was 50% (**Figure 1B**), with a median RFS time of 5.1 (0.6–29.3) months. In refractory and relapsed patients, the 1-year estimated OS rates were 25% and 33.3%, respectively (*P*=0.93; **Supplementary Figure S2**), and 1-year cumulative incidence of leukemia relapse was 47.5%.

Among 9 patients with allogeneic HSCT, 4 patients (44.4%) survived, and the median remission time was sustained for 505 (227–860) days. The remaining 5 patients died with a median time to death of 152 (52–255) days. The causes of death were related to relapse in 4 patients and severe pneumonia in 1 patient.

**Figure 2:**
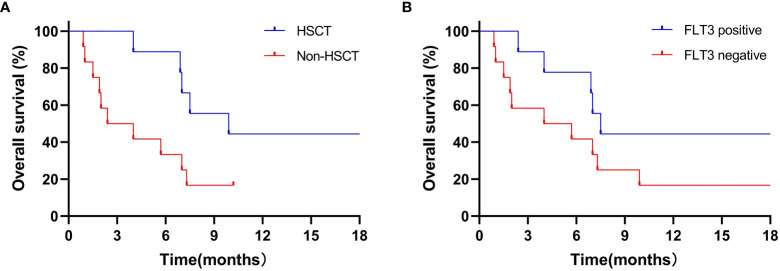
1-year estimated OS in transplantation patients **(A)** and in patients with FLT3 mutation **(B)**. **(A)** 1-year estimated OS rate was 44.4% in 9 patients who received transplantation, the median OS was not reached to investigation point in non-transplanted patients (*P*=0.03). **(B)** 1-year estimated OS rates was 44.4% in 9 FLT3-mutation patients, while 16.7% in 12 non-FLT3 mutation patients (*P*=0.12).

In 9 patients receiving transplantation, 1-year estimated OS rate was 44.4% (**Figure 2A**) with a median OS of 9.9 (4.0–30.1) months. The median OS was not reached in non-transplanted patients (*P*=0.03).

To compare the impact of CR/CRi on survival after transplantation, 4 patients with CR/CRi and 5 with PR/NR were treated with allogeneic transplantation, and they achieved 1-year estimated OS rates of 75% and 20% (*P*=0.10), respectively. Unfortunately, the relapse rate in patients with PR/NR after transplantation was high (40% *vs* 25%, *P*=0.03).

Of note, the 1-year estimated OS rates in the 9 patients with FLT3 mutation and 12 patients without FLT3 mutation were 44.4% and 16.7% (*P*=0.12; **Figure 2B**), with a median OS of 7.5 (2.4–30.1) and 4.9 (0.9–21.1) months, respectively. It is well known that CR/CRi after induction therapy has a better impact on the survival of patients with R/R AML. Hence, in patients with FLT3 mutation who had achieved CR/CRi, the 1-year estimated OS rate reached 66.7%. By contrast, patients who had not achieved CR/CRi did not reach the investigation endpoints (*P*=0.04).

## Discussion

Refractory or relapsed AML remains a major cause of death in patients with AML. However, it has been reported that patients with R/R AML treated with allogeneic HSCT in the second CR had a significantly better 5-year OS than those without receiving allogeneic HSCT (42% *vs* 16%, *P*<0.01) *(* 39).

The improved outcomes in R/R AML were mainly attributed to the reduced relapse rate of AML by allogeneic HSCT. It is well known that the elimination of minimal residual disease of leukemic cells prior to allogeneic HSCT plays a critical role in preventing AML relapse after transplantation. For instance, it has been reported that tumor burden prior to allogeneic transplantation is strongly associated with a high risk of relapse and negatively impacts long-term outcomes following allogeneic HSCT (40, 41). Moreover, Duval et al. (7) found that patients with CR before transplantation had higher OS rates. Therefore, CR achievement during R/R AML induction can reduce minimal residual disease prior to transplantation, which reduces relapse rates and improves long-term OS.

In our study, the ORR was 52.4%, and the CR rate was 38.1% after a single course of BHA, which is a higher response rate compared to that achieved with HiAra-C, CLAG, FLAG, or azacitidine combined with venetoclax (8, 11, 13, 42). This indicates a higher antileukemic efficacy of the BHA regimen.

Interestingly, patients with FLT3 mutations had strikingly higher CR/CRi rates than those without FLT3 mutations (66.7% *vs* 16.7%, *P*=0.03). A high CR/CRi rate was not associated with a refractory or relapsed state or the percentage of leukemic blasts in the bone marrow. This suggests that the BHA regimen is more effective in FLT3-mutated AML. Importantly, FLT3-mutated AML accounts for approximately 30% of all AMLs (43), with a 5-year relapse rate of 64%, resulting in a lower OS rate of 32% (44). The elevated CR/CRi rate gained from the BHA regimen certainly paved a safe way to conduct allogeneic HSCT in patients with FLT3 mutation, ultimately prolonging their survival.

Although FLT3 inhibitors have shown high remission rates in the treatment of AML with FLT3-ITD mutations, the problems of short-lasting remissions and the emergence of drug resistance in the short term have never been resolved. Regarding R/R AML with FLT3 mutations, inhibitors of mutated FLT3 such as sorafenib alone or in combination with chemotherapy can improve both response rate and long-term survival, so these FLT3 inhibitors nowadays act as a targeted drug for FLT3-mutated AML. However, FLT3 inhibitors only suppress the function of mutated FLT3 proteins but cannot degrade FLT3-ITD and FLT3-TKD oncoproteins. It has been described elsewhere by Larrue C et al. that proteasome inhibitor bortezomib is involved in degradation of FLT3-ITD oncoprotein *via* activation of autophagy, which leads to a cytotoxic apoptosis of leukemia cells *in vitro* (45). Moreover, Lam et al. found that homoharringtonine had a preferential antileukemia effect against FLT3-mutated AML cells *in vitro*, which was mediated by an inhibition of protein synthesis and a reduction in short-lived proteins including the total and phosphorylated forms of FLT3 and its resultant downstream signaling proteins (43). Therefore, bortezomib and homoharringtonine may play an enhanced role in killing leukemia cells, especially with addition of cytarabine. At this scenario, BHA may serve as a beneficial chemotherapy regimen for FLT-3 mutated R/R AML. Of note, degradation of PML/RARalpha oncoprotein by arsenic trioxide enables acute promyelocytic leukemia (APL) a curable leukemia, because arsenic trioxide can eradicate APL leukemic stem cells by degradation of the oncoprotein (46). Similarly, we deduced that the degradation of FLT3 mutation oncoprotein involved with bortezomib may also eradicate FLT3-mutated leukemia cells, and then it will achieve better therapeutic effects when combined with other drugs, such as FLT3 inhibitor or chemotherapy agents.

Three patients carrying FLT3 mutations have not obtained CR/CRi, among whom two carried two FLT3-ITD insertions, and one carried FLT3-ITD mutation concomitant with DNMT3A. These data indicated that different two FLT3 mutation sites might be barrier to a successful CR/CRi. This hypothesis is confirmed by the study of Rücker et al (47), who reported that more than one FLT3-ITD insertion site was identified as a significant unfavorable factor for CR, and the more insertion bit points existed, the less likely it was to achieve CR. It was also reported that DNMT3A mutations were associated with adverse outcomes among patients with FLT3 mutations (48); moreover, the presence of two concomitant DNMT3A mutations was associated with an independent adverse prognostic effect on OS in patients with AML (49). The FLT3-ITD mutation concomitant with DNMT3A double site mutations may be the underlying basis for ineffectiveness of BHA in the present study.

Although the 1-year estimated OS rate in patients with FLT3 mutation was higher than that in patients without FLT3 mutation, this difference was not statistically significant. We deduced that most patients with FLT3 mutation who achieved CR/CRi had not been treated with allogeneic transplantation, so primarily the induction therapy led to CR/CRi, but the various subsequent chemotherapies cannot ensure a prolonged survival and provided no OS advantage for patients with FLT3 mutation over those without.

In this study, the 1-year estimated OS was significantly higher in transplanted than in non-transplanted patients, indicating that allogeneic HSCT can improve R/R AML outcomes. Unfortunately, only 4 patients with CR/CRi and 5 with NR/PR received haploidentical bone marrow transplantation, which resulted in a lower OS than reported in the literature (39). Furthermore, the 1-year estimated OS after transplantation was higher in patients with CR/CRi than in patients with PR/NR, and the relapse rate was lower in the CR/CRi group than in the PR/NR group. These results suggest that a lower leukemia cell burden prior to transplantation enabled a reduced risk to relapse post transplantation (40). Collectively, the achievement of CR/CRi with BHA provided a time window to conduct allogeneic transplantation, thereby yielding a better survival in R/R AML, particularly in patients with FLT3-mutated AML.

BHA regimen had acceptable adverse events. Although grade 3/4 infections occurred in 90.5% of patients, all infections were quickly controlled by anti-microbial drugs. The mortality was 4.8% within 30 days, which was lower compared to 6.6–17.0% reported in patients with R/R AML in other studies (8, 10, 50). The high incidence of hypoalbuminemia, hypokalemia, and hypocalcemia, possibly related to poor diet due to chemotherapy, recovered quickly after BHA discontinuation. Three patients experienced grade 1/2 peripheral neuritis, but no patient had grade 3/4 neuritis, which presented no correlation with bortezomib.

Collectively, BHA regimen may be an option for treating R/R AML harboring FLT3 mutation. However, this trial has a limitation that the number of recruited patients was small, so a prospective trial should be conducted in order to certify that BHA will be effective for R/R AML carrying FLT3 mutation.

## Conclusion

In summary, BHA is a well-tolerated and effective induction chemotherapy with an ORR of 52.4% and a CR/CRi of 38.1% in patients with R/R AML. It achieved a higher CR/CRi of 66.7% in R/R AML patients carrying FLT3-ITD or FLT3-TKD mutations, suggesting BHA may be an option for R/R AML, particularly for FLT3-mutated those.

## Data availability statement

The datasets presented in this study can be found in online repositories. The names of the repository/repositories and accession number(s) can be found below: BioProject, accession number PRJNA937005.

## Ethics statement

The studies involving human participants were reviewed and approved by the Institutional Review Board and Ethics Committee of the Second Hospital of Dalian Medical University. The patients/participants provided their written informed consent to participate in this study.

## Author contributions

JY designed the clinical trial, guided and organized the performance of the clinical trial, and edited the manuscript. CZ performed the clinical trials, collected and analyzed the clinical data, wrote, and edited the manuscript. DG performed the clinical trials, collected and analyzed the clinical data, and data curation. XW, XS and YYan performed the clinical trials and data curation. YYang and JZ participated in the guidance of the data analysis and edited the manuscript. All authors contributed to the article and approved the submitted version.
