# Supplementary material for: Effectiveness of chemotherapy using bortezomib combined with homoharringtonine and cytarabine in refractory or relapsed acute myeloid leukemia: a phase II, multicenter, prospective clinical trial
Source: Front Oncol. 2023 Aug 18;13:1142449. doi: 10.3389/fonc.2023.1142449 (PMC10472935; doi:10.3389/fonc.2023.1142449)
Supplement: Supplementary file 1 [file DataSheet_1.docx]

Supplementary Material

**Effectiveness of chemotherapy using bortezomib combined with homoharringtonine and cytarabine in refractory or relapsed acute myeloid leukemia: A phase II, multicenter, prospective clinical trial**

**Chengtao Zhang^1†^, Da Gao^2†^, Xiaohong Wang^3^, Xiuli Sun^4^, Yan Yan^5^, Yan Yang^1*^, Jingjing Zhang^1*^, Jinsong Yan^1*^**

**Correspondence:**

Jinsong Yan(yanjsdmu@dmu.edu.cn); Jingjing Zhang(jingzhangbmt@126.com) ; Yan Yang ([yangyanxyfs@sina.com](mailto:yangyanxyfs@sina.com))

##
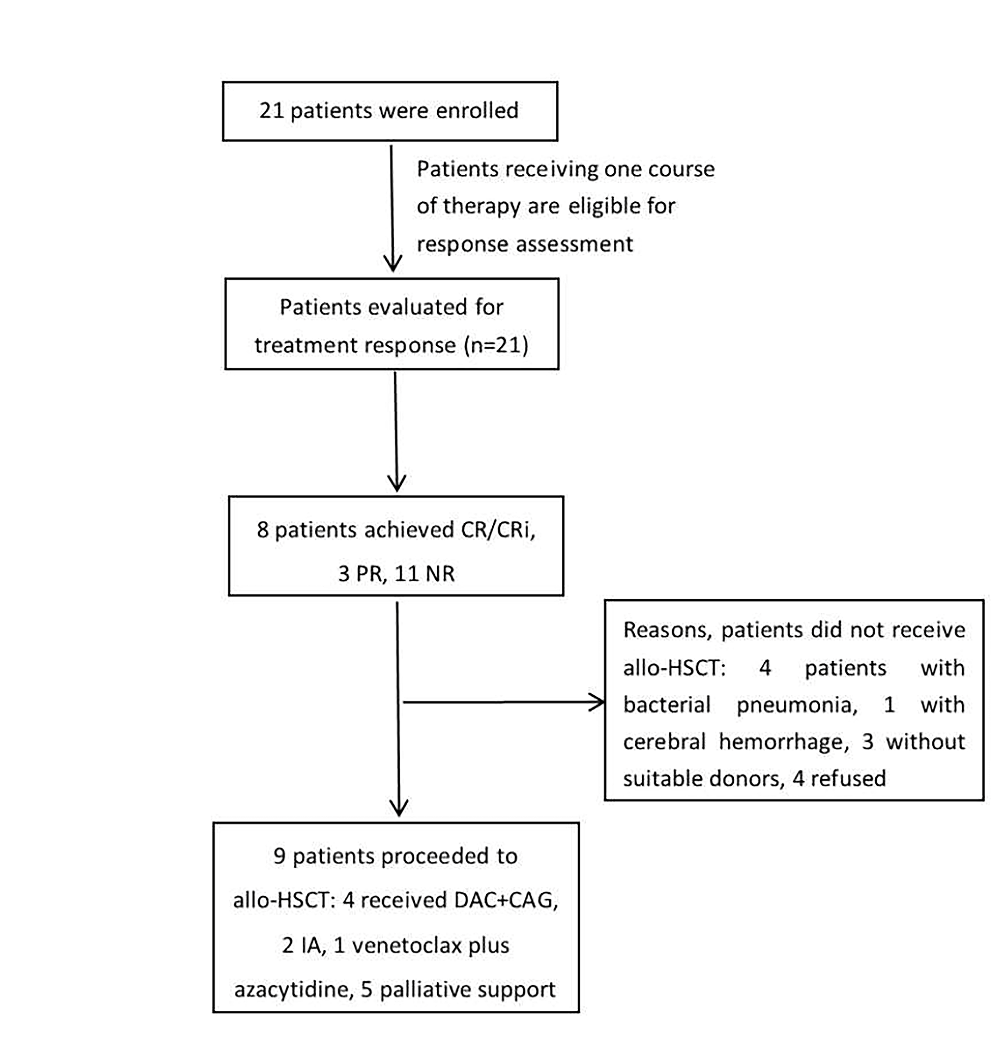


**Supplementary Figure 1.** Treatment scheme.

Abbreviations: allo-HSCT, allogeneic hematopoietic stem cell transplantation; CR, complete response; CRi, CR with incomplete hematologic recovery; PR, partial remission; NR, no remission; DAC, decitabine; CAG, cytarabine, aclacinomycin, granulocyte colony-stimulating factor; IA, intermediate-dose cytarabine and idarubicin.

**Table S 1.** Karyotyping and genetic mutations of the enrolled patients.

| **Patient No.** | **gene mutations** | **Karyotype** | **Response** |
| --- | --- | --- | --- |
| 1 | NRAS | 46,XX,-5,del(7)(q31q36),+10/46,XX | PR |
| 2 | **FLT3-ITD^low^**, DNMT3A | 46,XY | CRi |
| 3 | IDH2, NPM1, NARS | 46,XY | NR |
| 4 | U2AF1, IDH1, ASXL1, RUNX1 | 47,XY,+8,+15,-19 | NR |
| 5 | NARS, SF3B1 | 46,XX,t(8;X)(q22;q28) | NR |
| 6 | **FLT3-ITD^low^**, ZRSR2, MPL | 46,XY,der(3),add(16)(q24) /46,XY | CRi |
| 7 | **FLT3-ITD^high^(two insertion sites)**, NPM1, NRAS | 46,XX,add(9)(q34)/46,XX | NR |
| 8 | NPM1, CBL, ASXL1, TET2 | 46,XY | NR |
| 9 | PHF6 | 45,XY,-17/45,idem,+Y,del(5)(q13),+19/46,XY | PR |
| 10 | NPM1 | 48～51,XY,+X,inv(3)(q21;q26.2),del(6)(q12;p13),del(12)(p12;p13),  +del(12)(p12;p13),+15,+21/50,XY,+X,+8,+11,+16/46,XY | NR |
| 11 | TP53, STAG2, ZRSR2 | 46,XX,-4,t(8;21)(q22;q22),+mar/46,XX | CR |
| 12 | CEBPA, DNMT3A | 46,XY,add(12)(p13)/46,XY | CR |
| 13 | SRSF2, TET2, ASXL1 | 46,XY | NR |
| 14 | ETV6, ASXL1 | 46,XY/47,idem,+8/46,idem,del(1)(q31) | NR |
| 15 | **FLT3-TKD**, IDH1, NPM1 | 46,X,inv(X)(p22;q22),t(9;17)(p10;p10)/46,XY | CR |
| 16 | CEBPA, DNMT3A, NRAS | 46,XY | PR |
| 17 | **FLT3-ITD^low^**, **DNMT3A(double-locus)**, NPM1 | 46,XY | NR |
| 18 | **FLT3-TKD**, KIT, RAD21 | 46,XY,t(8;21)(q22;q22)/46,XY | CR |
| 19 | **FLT3-ITD^low^(two insertion sites)** | 45,XX,-7,t(9;11)(p21.3;q23.3),add(19)(p13)/46,XX | NR |
| 20 | **FLT3-ITD^low^, FLT3-TKD,** RUNX1, KRAS, SRSF2 | 46,XY | CRi |
| 21 | **FLT3-ITD^high^**,NPM1 | 45,XX,-19 | CR |

Abbreviations: Low, low allelic ratio (<0.5); High, high allelic ratio (≥0.5); CR, complete remission; CRi, CR with incomplete hematologic recovery; PR, partial remission; NR, no remission.

**Table S 2**. Treatment before or after BHA therapy and survival.

| **Patient No.** | **Before BHA**  **(number of courses)** | **After BHA**  **(number of courses)** | **Follow-up time (month)** |  | **Cause of death** | **Outcome** |  |
| --- | --- | --- | --- | --- | --- | --- | --- |
| 1 | DA, CLAG | Palliative support | 7.0 |  | Relapse | Death |  |
| 2 | DA, DAC+FLAG | allo-HSCT | 7.5 |  | Relapse | Death |  |
| 3 | DA, CLAG, CLAGM, MA, EA,  HA, DAC+FLAG | Palliative support | 1.0 |  | Severe pneumonia | Death |  |
| 4 | DA, DAC+FLAG | allo-HSCT | 4.0 |  | Severe pneumonia | Death |  |
| 5 | DA, DAC+FLAG | Palliative support | 1.5 |  | Disease progression | Death |  |
| 6 | IA^1^, DAC+FLAG | allo-HSCT | 30.1 |  | – | Alive |  |
| 7 | IA^1^, Sorafenib+IA^1^ | allo-HSCT | 7.0 |  | Relapse | Death |  |
| 8 | IA^1^, HA | DAC+CAG,  venetoclax+azacytidine | 2.0 |  | Disease progression | Death |  |
| 9 | TA, MA, Intermediate dose cytarabine(3), DAC+CAG(7) | allo-HSCT | 21.1 |  | – | Alive |  |
| 10 | IA^1^(2) | Palliative support | 0.9 |  | Cerebral hemorrhage | Death |  |
| 11 | DA, DAC+HA | allo-HSCT | 17.7 |  | – | Alive |  |
| 12 | DA(2), DAC+HAG, CLAG | IA^2^ | 1.9 |  | Sepsis | Death |  |
| 13 | DA, DAC+FLAG | DAC+CAG | 5.7 |  | Disease progression | Death |  |
| 14 | DA, High-dose cytarabine(2) | allo-HSCT | 9.9 |  | Relapse | Death |  |
| 15 | HAA(2)、IA^2^、  High-dose cytarabine(2) | allo-HSCT | 10.8 |  | – | Alive |  |
| 16 | IA^1^, DAC+HAG,  venetoclax + DAC(3) | DAC+CAG, EA | 7.3 |  | Disease progression | Death |  |
| 17 | IA^1^, Sorafenib +IA^1^ | DAC+CAG | 4.0 |  | Disease progression | Death |  |
| 18 | HAD, Sorafenib +HAD | IA^2^ (2) | 10.2 |  | – | Alive |  |
| 19 | IA^1^, Sorafenib+DAC+FLAG | allo-HSCT | 6.9 |  | Severe pneumonia | Death |  |
| 20 | DA, Sorafenib, DAC+ venetoclax | Venetoclax+azacytidine | 2.4 |  | Cerebral hemorrhage | Death |  |
| 21 | IA^1^ | Palliative support | 8.3 |  | – | Alive |  |

Abbreviations: DA, regimen of daunorubicin and standard-dose cytarabine; IA^1^, regimen of idarubicin and standard-dose cytarabine; IA^2^, regimen of idarubicin and intermediate-dose cytarabine; CLAG, regimen of cladribine, cytarabine, granulocyte colony-stimulating factor; FLAG, regimen of fludarabine, cytarabine, granulocyte colony-stimulating factor; CLAGM, regimen of cladribine, cytarabine, granulocyte colony-stimulating factor and mitoxantrone; MA, regimen of mitoxantrone and intermediate-dose cytarabine; EA, regimen of etoposide and intermediate-dose cytarabine; HA, regimen of homoharringtonine and intermediate-dose cytarabine; DAC, decitabine; TA, regimen of pirarubicin and standard-dose cytarabine; CAG, regimen of aclarubicin, low-dose cytarabine and granulocyte colony-stimulating factor; HAG, regimen of homoharringtonine, low-dose cytarabine and granulocyte colony-stimulating factor; HAA, regimen of homoharringtonine, standard-dose cytarabine and aclarubicin; HAD, regimen of homoharringtonine, standard-dose cytarabine and daunorubicin; allo-HSCT, allogeneic hematopoietic stem cell transplantation.


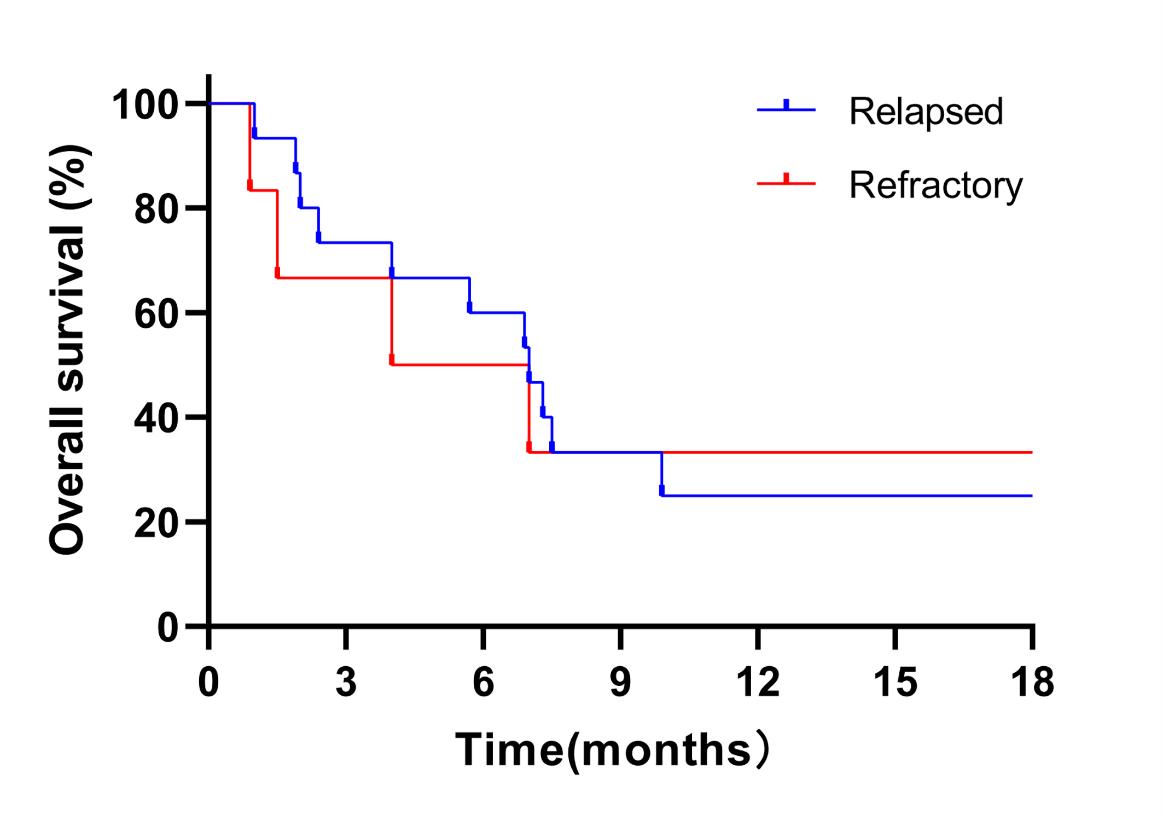


**Figure S 2.** The 1-year estimated overall survival rate for patients with refractory or relapsed AML.

**Figure legend**

**Figure S1**

**Figure S1.** Treatment scheme.

The Figure S1 indicated the treatment scheme.

**Figure S2**

**Figure S2.** 1-year estimated overall survival rate for patients with refractory or relapsed AML.

1-year estimated OS rates were 25% in patients with refractory AML and 33.3% in ones with relapsed AML (*P*=0.93).
